# Cellular and Antibody Based Approaches for Pediatric Cancer Immunotherapy

**DOI:** 10.1155/2015/675269

**Published:** 2015-10-26

**Authors:** Michael A. Huang, Deepa K. Krishnadas, Kenneth G. Lucas

**Affiliations:** Department of Pediatrics, Hematology/Oncology, University of Louisville, Louisville, KY 40202, USA

## Abstract

Progress in the use of traditional chemotherapy and radiation-based strategies for the treatment of pediatric malignancies has plateaued in the past decade, particularly for patients with relapsing or therapy refractory disease. As a result, cellular and humoral immunotherapy approaches have been investigated for several childhood cancers. Several monoclonal antibodies are now FDA approved and commercially available, some of which are currently considered standard of practice. There are also several new cellular immunotherapy approaches under investigation, including chimeric antigen receptor (CAR) modified T cells, cancer vaccines and adjuvants, and natural killer (NK) cell therapies. In this review, we will discuss previous studies on pediatric cancer immunotherapy and new approaches that are currently being investigated in clinical trials.

## 1. Introduction

Each year there are an estimated 15,780 children (age less than 19 years) who are diagnosed with cancer in the United States [1] and approximately 250,000 children worldwide [2]. While use of chemotherapy and radiation approaches has resulted in improved cure rates, cancer remains the most common cause of disease-related mortality in America. Children with relapsing or therapy refractory cancer have limited treatment options with further intensification of chemotherapy or radiation. With the additive toxicities of conventional treatment approaches and limited efficacy in achieving cure, many pediatric immunotherapy studies have targeted patients with relapsing cancer in a Phase I setting, with a long range goal of using immune-based therapy to prevent relapse or treat minimal disease.

Ongoing challenges in pediatric cancer immunotherapy include identifying subjects who may be able to benefit from this approach, since many of these patients have significant immunocompromise from previous therapy, and have limited ability to achieve an immune response to target antigens. For this reason, there has been much interest in the use of adjuvant agents in the setting of cancer vaccines, adoptive cellular immunotherapy, and the use of monoclonal antibodies. Advances in technology over the past decade have resulted in increased understanding of cancers on a genomic level as well as identification of new tumor-associated antigens. This in turn has paved the way for the development of novel monoclonal antibody and cell-based immunotherapy agents. In this review, we will discuss immunotherapy with monoclonal antibodies (mAbs), dendritic cell (DC), and cancer vaccines, as well as cellular immunotherapy with NK cells, CAR T cells, and antigen specific cytotoxic T lymphocytes (CTL).

## 2. Monoclonal Antibodies

**Figure 1:**
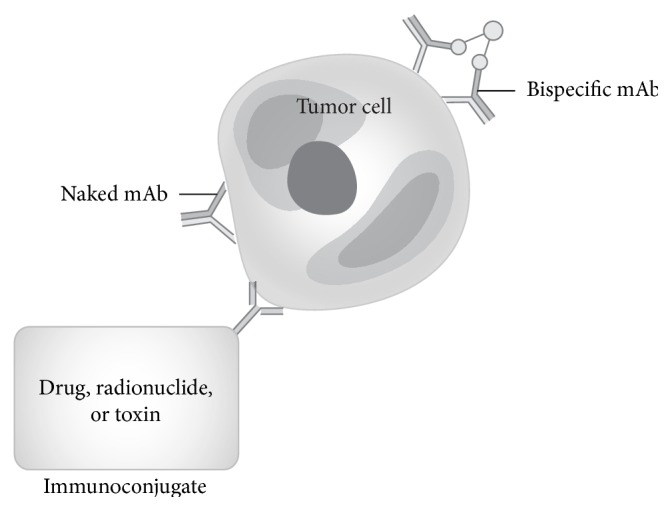
Different mechanisms of tumor cell killing by monoclonal antibody therapy. Monoclonal antibodies exhibit tumor cell cytotoxicity by targeting a specific tumor antigen. Immunoconjugates are monoclonal antibodies conjugated to drugs, toxins (immunotoxins), or radionuclides. mAb: monoclonal antibody.

mAbs work by binding to antigens on the tumor cell surface and either facilitating antibody-dependent cellular cytotoxicity (ADCC) by the host's immune system or more directly serving as a vector for a toxin or radionuclide (Figure 1). The main advantage of mAbs over cell-based approaches (e.g., CAR and tumor vaccines) is that they can be stored in clinic and hospital pharmacies and advanced expertise in cell-based therapeutics is not needed.

Rituximab is a mAb targeting CD20, an antigen expressed on B-cell lymphomas, and became the first ever mAb approved for clinical use in 1997. It is approved for use in non-Hodgkin lymphoma (NHL) as well as chronic lymphocytic leukemia. CD20 is present in virtually all patients with lymphocyte predominant Hodgkin lymphoma (LPHL) and in a significant minority of patients with classical Hodgkin lymphoma (HL). In one Phase II trial for LPHL, rituximab showed a 96% overall response rate, with 75% 1-year EFS [3]. This antibody has also been used successfully to treat B-cell lymphoproliferative disease and lymphomas following solid organ and stem cell transplantation [4]. While the use of anti-B-cell therapy often results in hypogammaglobulinemia, this is deemed relatively safe given the availability of gamma globulin replacement.

In 2011, brentuximab vedotin, an anti-CD30 mAb conjugated to monomethyl auristatin E, a microtubule inhibitor, was approved by the FDA for relapsing or refractory HL and anaplastic large cell lymphoma (ALCL). Overall response rates in several case reports of pediatric relapsing HL or ALCL showed a 47–64% overall response rate [5]. A Children's Oncology Group (COG) study is underway looking at administering brentuximab vedotin and both eliminating bleomycin (due to potential risk of increased pulmonary toxicity with concurrent use) and decreasing the cumulative dose of vincristine, another antimicrotubule agent.

In 2000, the FDA approved gemtuzumab ozogamicin (GO) for acute myelogenous leukemia (AML), an anti-CD33 mAb conjugated to the drug calicheamicin. The drug was later withdrawn from the market in 2010 due to concerns of hepatic sinusoidal obstruction syndrome and lack of statistically significant clinical benefit in an adult Phase III trial [6]. Subsequent studies have shown that lower doses of GO than previously used could be safely administered, leading to renewed interest in clinical studies with this agent [7]. Inotuzumab (CMC-544) is an anti-CD22 conjugate linked to ozogamicin which has shown activity in Phase II trials in pediatric B-cell ALL [8]. Studies are underway to better elucidate its role in refractory or relapsing pediatric B-ALL. Moxetumomab pasudotox is an anti-CD22 mAb conjugated to a pseudomonal exotoxin that is being investigated in pediatric B-cell ALL prior to allogeneic stem cell transplantation (SCT). An anti-CD22/anti-CD19 mAb agent is also undergoing clinical investigation and has shown promising results in a Phase I study for refractory or relapsing pediatric B-cell ALL [9]. mAbs conjugated to radionuclides including CHT-25 (anti-CD25 mAb conjugated to 131-iodine) and ibritumomab (anti-CD20 mAb conjugated to 90-yttrium) have shown efficacy in pediatric Hodgkin and non-Hodgkin lymphoma, respectively [10, 11]. Radionuclide immunoconjugates, however, can lead to prolonged cytopenias, limiting their use.

A new class of mAbs, called bispecific antibodies, are molecules that recognize two distinct antigens on the tumor cell surface. Blinatumomab is a bispecific T-cell engager (BiTE) that targets CD19 positive cells and simultaneously binds to CD3-positive, activated T cells for killing. This mechanism of action allows bypassing MHC Class I restriction to achieve killing by T cells. A COG Phase II study incorporating its use in both ALL and B-cell lymphoma is underway. There is also preclinical data supporting the possibility of targeting T cells against neuroblastoma with the use of 3F8BiAb, a bispecific antibody to GD2 (present on neuroblastoma cells) and CD3 (expressed on activated T cells) [12].

Monoclonal antibodies targeting the disialoganglioside GD2, which is expressed on tumors of neuroectodermal origin, have been in clinical trials for over two decades. GD2 is an ideal target for neuroblastoma since its expression is highly restricted on normal tissues, principally to the cerebellum and peripheral nerves. The chimeric mAb ch14.18 against GD2 has become the most widely used mAb in pediatric cancer and its use in the adjuvant setting following standard neuroblastoma therapy has resulted in improved survival for patients with metastatic disease. A pivotal study was performed by the COG in which there was an improved 2-year EFS of 64% (compared to 44% with cis-retinoic acid alone) when given with aldesleukin (IL-2) and granulocyte monocyte colony stimulating factor (GM-CSF) [13]. Immunotherapy with anti-GD2 has now become the standard of care for patients with metastatic neuroblastoma. Another anti-GD2 drug, humanized 14.18-3F8 conjugated to IL-2, has shown activity in Phase II trials in children with refractory/relapsing neuroblastoma [14]. GD2 is also highly expressed on osteosarcomas [15], and Phase I studies are underway to investigate its role in the therapy of this tumor.

Tumor signaling and growth pathways have also served as target antigens in pediatric solid tumors. Vascular Endothelial Growth Factor (VEGF) is a signaling protein that is critical for solid tumor vascular proliferation. Bevacizumab, a VEGF inhibitor, has shown activity both as a single agent and in combination with other chemotherapy agents for a variety of tumors, including recurrent low grade glioma [16, 17], medulloblastoma [17, 18], neuroblastoma, rhabdomyosarcoma, Wilms tumor, and hepatocellular carcinoma [19]. Cetuximab is a mAb directed against Epidermal Growth Factor (EGF) receptor and although it is approved for several adult malignancies, its role and potential benefit in pediatric solid tumors are still being investigated. Human Epidermal Growth Factor Receptor 2 (HER2) expression, which is typically considered a biomarker for breast cancer, has been associated with poor outcome in osteosarcoma. The use of trastuzumab, an anti-HER2 mAb, did not result in significant differences in EFS and OS when studied in conjunction with standard chemotherapy in patients with metastatic osteosarcoma [20]. The Insulin Growth Factor-1 receptor (IGF-1R) pathway has been the target of various mAbs, but clinical efficacy has been variable. The administration of figitumumab, an anti-IGF-1R mAb, has been associated with objective responses in Ewing sarcoma patients [21], whereas R1507, another IGF-1R antagonist mAb, had mixed results in two studies [22, 23]. Racotumomab, an anti-idiotype vaccine targeting NeuGcGM3, when tested in children (Phase I study) with relapsing or resistant neuroblastoma and other tumors expressing NeuGcGM3, showed IgM and IgG response in most patients [24].

Monoclonal antibodies that target the T-cell inhibitory checkpoints are also undergoing investigation for pediatric solid tumors. Programmed cell death receptor (PD1) is a cell surface receptor that plays an important role in downregulating T-cell activation, which in turn leads to tumor tolerance. Cytotoxic T lymphocyte-associated protein 4 (CTLA-4) is another protein receptor that functions as an immune checkpoint and helps downregulate the immune system. Nivolumab, an anti-PD1 mAb, and ipilimumab, an anti-CTLA-4 mAb, are FDA approved for the treatment of melanoma. Nivolumab with or without ipilimumab is being investigated for the treatment of recurrent or refractory pediatric solid tumors.

## 3. Chimeric Antigen Receptor T Cells

**Figure 2:**
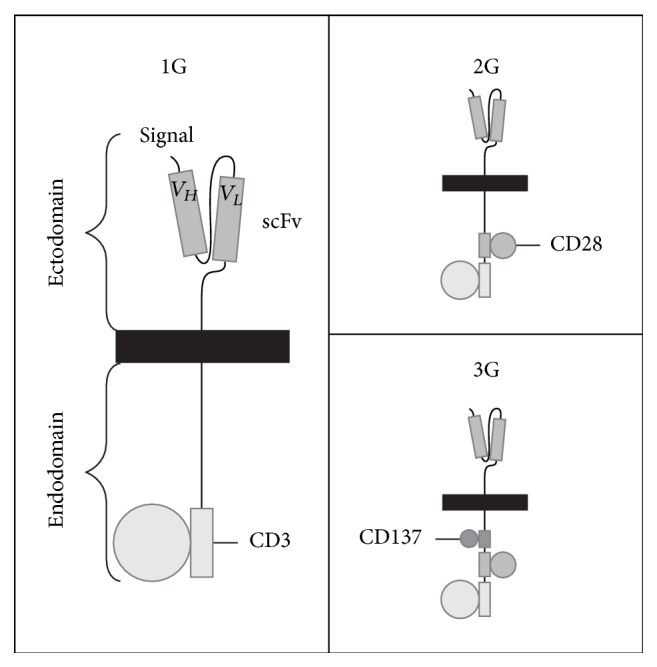
Different generations of chimeric antigen receptors (CARs). Left to right, top to bottom. First-generation CARs consist of scFv fragment against a tumor antigen (e.g., GD2 in neuroblastoma, CD19 in B-cell malignancies) linking a CD3 signaling chain. Second- and third-generation CARs incorporate 1 or 2 costimulatory molecules, respectively (e.g., CD28, CD137). 1G: first generation; 2G: second generation; 3G: third generation.

Recent advances in cell culture and manipulation technology have resulted in the ability to expand clinically relevant numbers of engineered T cells that express chimeric antigen receptors (CARs). CARs are genetically engineered receptors that redirect T cells to a selected tumor antigen (Figure 2). Cancer cells often escape T-cell immune surveillance by downregulating HLA molecules involved in antigen presentation. The main advantage of CAR T cells is that this approach bypasses the need for tumor antigen presentation to MHC Class I molecules, hence providing the dual benefit of target specificity akin to mAbs and the killing capacity of CTL. Autologous T cells are collected from the patient and subsequently expanded; CARs are then genetically inserted into those T cells using viral vectors, DNA transposons, or RNA transfection [25]. These CAR T cells can later be reinfused to the patient to treat refractory malignancies. First-generation CARs consist of a single Fv fragment or activation domain against a tumor-associated antigen. Second- and third-generation CAR T cells involve the addition of one or two costimulatory molecules (e.g., CD28 and CD137), respectively. The addition of a costimulatory molecule in second-generation CAR T cells has led to demonstrable improvement in T-cell proliferation and survival [26].

There are at least 30 studies on https://clinicaltrials.gov/ involving CAR T cells that allowed for pediatric enrollment. Less than a third were designed for solid malignancies, with the majority aimed at CD19+ hematologic malignancies. Sustained complete remissions were attained in adults with B-cell ALL, NHL, and refractory CLL by targeting CD19 [27–29]. Based on the dramatic responses noted in adults, CD19 CAR T-cell infusions were performed in pediatric ALL with similar outcomes. In the first Phase I trial, 2 children with refractory, heavily pretreated B-cell ALL achieved complete remissions, with one relapsing from CD19-negative disease 2 months after CD19 CAR T-cell therapy [30]. A study from Children's Hospital of Philadelphia reported outcomes in 25 children receiving CAR T cells, the majority of whom received a prior allogeneic SCT. Complete remission was achieved in up to 90% of patients, which included 2 patients previously treated with blinatumomab. Event-free and overall survival at 6 months was 67% and 78%, respectively, and durable remissions up to 24 months were observed [31]. CARs targeting other lymphoid (e.g., CD22, CD30) and myeloid (e.g., CD13, CD33) antigens are currently in development. B-cell aplasia leading to prolonged hypogammaglobulinemia is a concern with CD19-targeted CAR T-cell therapy. In one study which looked at CD19-targeted CAR T-cell therapy in 21 children and young adults (age <30 years), no cases of prolonged B-cell aplasia were observed [32]. Trials looking at targeting CD30 and CD33 for other hematologic malignancies are ongoing.

There is limited data with use of CAR T cells for malignant solid tumors. In a Phase I study using first-generation CAR T cells targeting GD2 for refractory neuroblastoma, 27% of those with active disease at time of the anti-GD2 CAR T-cell infusion subsequently went into complete remission, with 2 durable remissions >60 months [33]. Another first-generation CAR T-cell study targeting tumor-associated CD171, an L1 cell adhesion molecule, did not show clinical efficacy. CAR T cells are being included in clinical trials targeting IL-13R alpha, which is expressed on gliomas and medulloblastomas not on normal CNS tissue, GD2 for osteosarcoma, and HER2, which is highly expressed in osteosarcoma and some cases of medulloblastoma.

Despite success with CAR T cells, there are significant potential toxicities associated with this therapy. Cytokine release syndrome is a known side effect from the CAR T-cell infusion but is reversible with the benefit of tocilizumab, an anti-inflammatory agent against IL-6 [34]. Severe, life-threatening cytokine syndrome can occur in up to 27% of patients [31]. One patient expired from respiratory distress in a Phase I study in metastatic HER2 cancer patients using third-generation HER2 CAR T cells, and this was ascribed to T cells recognizing low levels of HER2 in lung tissue [35]. Another concern is that long term follow-up may possibly reveal unexpected toxicities from CAR T-cell therapy. Conversely, CD19-directed CAR T cells in B-cell leukemias lead to agammaglobulinemia, a condition easily corrected with gammaglobulin replacement. Targeting cancer germline antigens (e.g., NY-ESO-1, MAGE) is another viable option as these antigens are expressed by healthy cells only during fetal development and not later in life [36]. Methods to minimize or eliminate the negative side effects of CAR T cells include decreasing T-cell doses, the use of suicide gene systems (e.g., HSV-TK, drug inducible caspase-9), and incorporation of defined surface antigens (e.g., CD20) that could be later targeted with mAbs [37].

## 4. Cell-Based Immunotherapy and Tumor Vaccines

Several T-cell-based immunotherapy strategies are under investigation, such as autologous/allogeneic transplantation of tumor specific CTLs, oncolytic virotherapy, allogeneic NK cell infusions, and tumor vaccines [33, 38]. Adoptive immunotherapy with autologous and/or allogeneic cancer antigen specific CTL has been investigated in both solid and hematologic cancers [39]. Oncolytic virotherapy uses attenuated viruses targeted to specifically infect host cancer cells leading to direct antitumor effects and immunologic cell death from tumor antigen presentation [40]. Killer immunoglobulin- (KIR-) mismatched NK cell infusions are currently under investigation for pediatric leukemias, neuroblastoma, and sarcomas [41, 42]. Several tumor vaccine approaches have also been studied in pediatric cancer, using peptide alone or DC pulsed with tumor peptides or lysates.

EBV-associated posttransplant lymphoproliferative disease (PTLPD)/lymphoma was treated by infusing donor-derived EBV-specific CTL generated using EBV-transformed lymphoblastoid B-cell lines. Infusion of EBV-specific T cells after SCT was found to be highly effective to prevent the development of PTLD and treat preexisting disease [43–45]. EBV-associated tumors express viral antigens and can be targeted using EBV-specific CTL. The association of pediatric nasopharyngeal carcinoma with EBV makes EBV antigens an immunotherapeutic target for cell-based therapy. Several ongoing and recently completed trials utilize either autologous or most closely HLA-matched EBV-specific (LMP-1 and LMP-2) CTL to treat nasopharyngeal carcinoma (NCT00953420, NCT01447056, and NCT00516087).

## 5. NK Cell-Based Immunotherapy

The antitumor effects of NK cells make them a potential immunotherapy option in the postallogeneic hematopoietic transplant setting and also in the nontransplant setting. Adoptive therapy with NK cells has been carried out to treat AML and several solid tumors, including ovarian cancer, melanoma, breast cancer, renal cell cancer, and advanced lung cancer [46–49]. Due to relatively low numbers in the peripheral blood, immunotherapy with NK cells requires ex vivo expansion to achieve clinically relevant numbers, and, during the expansion process, these cells display increased expression of activation markers, chemokine receptors, and adhesion molecules [50–52]. Further, these studies using ex vivo activated and expanded NK cells have demonstrated extensive cytotoxicity against various tumor cells without affecting the healthy cells. Two pediatric trials (NCT01875601 and NCT01944982) employing ex vivo activated and expanded allogeneic NK cells are ongoing and one trial (NCT00640796) has been recently completed (in 2014). A pilot study on 10 children with AML employed the use of nonactivated, KIR/KIR ligand mismatched haploidentical donor NK cells along with exogenous IL-2 with all 10 subjects remaining in remission 2 years after infusion [53]. A similar study using NK cells in adults reported complete remission in 75% of subjects with KIR/KIR ligand mismatches [48, 54]. There are more than 20 open or recently completed clinical trials employing NK cell-based immunotherapy for pediatric cancers as reported recently in a comprehensive review by McDowell and coworkers [54]. These studies either employ NK cells as monotherapy or in combination with chemotherapy and/or a mAb, such as anti-GD2 antibody. A recent study by Rubnitz and coworkers reported that 76% of children with relapsing or refractory leukemia treated with chemotherapy followed by the infusion of haploidentical NK cells proceeded to hematopoietic cell transplantation and 31% were alive when compared to a parallel study conducted by the same group with only 13% of patients alive with the same chemotherapy, but without NK cells [55, 56].

## 6. Tumor Vaccines

A major challenge of cancer vaccines is the fact that standard chemotherapy agents can be highly immunosuppressive, limiting the ability of patients to respond to the vaccine [57]. Immunologic adjuvants such as toll-like receptor (TLR) agonists have been used clinically to facilitate both antigen presenting cell and responder cell functions [58, 59]. Another problem is identifying appropriate tumor antigens to target in a vaccine, based on expression patters in individual malignancies. While optimal antigens have not been defined for many pediatric cancers, several MHC-restricted cancer antigens have been identified on pediatric tumors, of which cancer germline antigens (CGAs) are the most well studied [31]. The earliest evidence for the safety and potential efficacy of cancer vaccines was in malignant melanoma after targeting CGA [60]. Downregulation of MHC Class I and tumor specific antigens is a common mechanism of tumor immune escape, and some highly immunogenic CGAs can be epigenetically upregulated with exposure to demethylating agents (e.g., decitabine, azacytidine). Our group reported the epigenetic upregulation of MAGE-A1, MAGE-A3, and NY-ESO-1 antigens on neuroblastoma and sarcoma cells after exposure to decitabine, thereby enhancing the recognition of tumor cells by antigen-specific CTLs [61, 62].

There are several potential options for cancer vaccines including DC pulsed with tumor lysate, whole tumor proteins, HLA-restricted peptide antigens, and overlapping whole tumor antigen peptide mixes, with or without adjuvants. In one study of children with high-grade glioma receiving vaccines with DC pulsed with tumor lysates, sustained remissions were demonstrated in children with minimal residual disease at the time of vaccination [63]. In another Phase I trial, a 20% response rate was noted with the use of tumor lysate pulsed DC for children with relapsing solid tumors [64]. In a study by Bowman and coworkers, adenovector-mediated transfer of the IL-2 gene into autologous neuroblasts in patients with relapsing neuroblastoma led to a clinically effective antitumor immune response mediated by both helper and cytotoxic T lymphocytes in some patients [65]. The same group later showed that an allogeneic tumor vaccine combining transgenic human lymphotactin with IL-2 in patients with advanced and refractory neuroblastoma led to 2-fold expansion of CD4^+^ T cells and 3.5-fold expansion of NK cells, inducing a more potent immunologic and clinical response. Twenty-eight percent of the patients had significant increase in NK cytolytic activity and 71% of the patients made IgG antibodies [66]. Currently, there are over 10 ongoing or recently completed vaccine trials for various pediatric solid tumors including pontine and high-grade glioma, medulloblastoma, neuroectodermal tumors, neuroblastoma, and different types of sarcoma [67].

At the author's institution, DC vaccine trials are open incorporating decitabine followed by DC/MAGE-A1, MAGE-A3, and NY-ESO-1 vaccine in the treatment of relapsing/refractory neuroblastoma, sarcomas, and brain tumors. We have previously published our Phase I DC vaccine experience with neuroblastoma and sarcomas wherein one of ten patients achieved a complete response. Two patients were disease-free at start of DC vaccine therapy of which one remains disease-free 2 years off from therapy [68].

## 7. Summary

There have been several new developments in immunotherapy over the past decade which have dramatically altered the clinical course of children with relapsing or otherwise high risk malignancies. The combination of ch14.18 antibody, aldesleukin, and GM-CSF is a prime example of how mAbs can have a significant impact on patient survival and the notion that immune strategies can safely be incorporated into our standard chemoradiation approach. CAR T-cell therapy shows promise but appropriate target antigens for other tumors besides relapsing pediatric ALL still need to be identified. Tumor vaccines have shown a modest response in some pediatric solid tumors, with better results noted in the setting of minimal residual disease burden and with the use of adjuvants.
